# Vaccination

**Published:** 1895-06

**Authors:** L. Hoopes

**Affiliations:** West Chester, Pa.


### VACCINATION.

L. HOOPES, M. D., WEST CHESTER, PA.

The question as to the prophylactic value of vaccination, as usually practiced, against small-pox, is one upon which there is difference of opinion in all schools of medicine.

While a student I was taught that it was unquestionably the proper thing to do, and that if not really prophylactic, it at least modified the disease so as to render it almost without danger to life.

With this idea I entered practice at Pottstown, Pa., in the summer of 1871. But the tranquillity of my mind on this point was destined soon to be disturbed. In the winter of 1871–2 a severe and very fatal epidemic of small-pox visited Philadelphia, and I soon discovered that my brothers of the old school were busy vaccinating their patients by the old arm-to-arm method, so I decided to improve on that, and announced that I would vaccinate with cow-pox virus and avoid the danger of transmitting disease from one person to another. My proposition seemed to take with the people, and I was patronized much beyond the limit of my, then, very meagre practice, for quite a number had suffered from the bad effects of vaccination in years gone by, and so, while feeling the urgent need of protection, and knowing no other means, they were glad to accept my proposition; and after my first week's work in that line, I felt that I had achieved a good thing and afforded protection to every one who had thus given me his confidence. But in a few days I was called to see the servant in a wealthy and influential family whom I had vaccinated. She was a robust colored woman about thirty-five years of age, who had never been sick in her life before. I found her suffering intensely from septic poisoning. The arm was enormously swollen, with no sign of a vaccine vesicle, and very painful. She had a raging fever, with delirium, and I thought she would surely have to lose her arm, if not her life. I felt that my reputation was tottering. But fortunately she recovered under strict homœopathic treatment.

This case set me to thinking that vaccination was a dangerous expedient—no better than small-pox itself. I watched the medical journals and newspapers to see what I could learn, and I soon found that, in Philadelphia, many families had been entirely swept away by the dread disease, where the attending physicians testified that they had been properly vaccinated and it had taken well, within three months. This was knocking the props from under the safeguard. Vaccination evidently did not prevent persons from being attacked by small-pox in its most malignant form.

Since that time I have been opposed to vaccination in the original way, although I have frequently done it under protest.

I believe that the introduction of foreign diseased matter immediately into the circulation is a very dangerous and unscientific thing to do, for are we not continually warned against allowing any foreign or diseased matter to come in contact with wounds owing to the danger of producing septic poisoning and death? Now in the ordinary manner of vaccination do we not deliberately make a wound and put into it foreign diseased matter for the ostensible purpose of preventing or modifying a disease with which the patient may never meet? Now if it is so dangerous in surgical cases, is it any less dangerous in vaccination?

Then there is the risk of transmission of disease from one person to another, or from the cow to the person. It is a well-known fact that syphilis has been widely spread by vaccination, as the following circumstance, noted in Erichsen's *Surgery*, fifth London ed., p. 525, will show: "Syphilis has also been spread widely among young children by vaccinating them with lymph from a syphilitic child. One of the most unquestionable of these accidents is that which occurred in the Subapenine Valley of Rivalta, in Piedmont, in 1861. Dr. Pacchiotti, of Turin, who was employed by the Italian Government to report on this attack, has published an account of it. The facts are shortly these: In May, 1861, an apparently healthy child, named Chiabrera, was vaccinated at Rivalta with lymph sent from Acqui for the purpose. Ten days after this vaccination—on June 7th—forty-six healthy children were vaccinated at one sitting from this child. Again, on the 12th of June, seventeen other healthy children were vaccinated from one of the forty-six. Thirty-nine of the first series of forty-six and seven of the second series of seventeen received syphilis with the vaccine disease, making a total of forty-six out of sixty-three children in a mountain village simultaneously inoculated with syphilis. Some months elapsed before the vaccination was suspected to have been the source of the children's bad health. By the 7th of October, when attention was drawn to this spreading disease, six of the forty-six syphilized children had died without receiving any treatment, fourteen were recovering, and three were in a precarious condition. Twenty-three were dispersed through the country, and their condition was unknown until further researches traced them out. In addition to the children twenty women suckling them were inoculated with syphilis from the children; through the mothers the disease had reached some of the husbands and even the elder children of the different families."

This is a *horrible* picture! Forty-six healthy children inoculated with a loathsome disease which will probably remain with them for life and make life miserable, besides their liability to transmit it to future generations! Will any one say this is preferable to risking small-pox? But my friends who believe in vaccination will say, "This is a circumstance which seldom happens." That is probably true of syphilis, now that we use bovine virus, but if one constitutional disease is communicable by vaccination, is not the same true of any other constitutional disease? Are not the constitutional diseases of the cow communicable in the same way? May not this account for the increase of tuberculosis, cancer, etc.? We know that tuberculosis and other diseases may remain latent through one or more generations, and in these cases we are unable to discover it except by family history; but, nevertheless, it is there, and, in my opinion, it is just as amenable to communication by vaccination as the syphilis in the apparently healthy child above mentioned. The propagators of bovine virus cannot *know*, except through family history for two or three generations back, whether there is latent tuberculosis or other disease in the heifer or not.

Constitutional diseases often lie dormant in some members of a family in which such a disease exists, and may remain so through life, if nothing disturb the constitution to arouse it into activity and bring it to the surface. Vaccination is very liable to stir up such latent disease through the efforts of Nature to rid herself of the vaccine disease, and in many cases no amount of care or treatment will suffice to cure or bring them back to the dormant condition, and the unfortunate patient is made miserable for the remainder of life, when, had the latent disease not been aroused into activity by vaccination they might have enjoyed fairly good health.

But I think all this trouble and risk may be avoided and the community enjoy as much or *more* protection from small-pox than they do under ordinary vaccination; for it is my opinion, from my own observation and from what I can learn from others, that both Vaccininum and Variolinum, when administered by the mouth, in potentized form, exert a decidedly protective influence against the disease, and the danger of injecting poisonous substances into the circulation is avoided; for it is well known that even rattlesnake poison may be taken into the stomach without danger, but injected into the circulation it is almost certain death.

About twelve years ago I treated a case of small-pox in a colored man who had six children, all of whom had been vaccinated a short time previous, but only two of them had taken. I gave orders that the children should not be allowed up-stairs; but every time I visited my patient I found some of them playing in his room, sometimes on the bed. The case presented a clear picture of the proving of Vaccininum, and that remedy was given to both father and children, in the 30th potency, one dose each day. The father made a rapid recovery and none of the children took the disease.

A year or two ago I read in a sample allopathic journal which was sent me, and which I have unfortunately lost as well as forgotten the name, of three allopathic physicians who had used vaccine virus diluted, as near as I could make out, to about our 3x potency, and administered it by the mouth. The writer of the article was one of the three. He said that from 1837 to 1842 the small-pox was very fatal, and he lost nearly all his cases until his son was taken with the disease, and in spite of all he could do he grew steadily worse and his speedy death seemed inevitable. Then he sat down in despair and after some serious thought he decided to give him diluted vaccine virus, which he did with immediate benefit, and the boy made a rapid recovery. He and two others of his colleagues gave it in every case from that time on and all recovered. They also gave it as a prophylactic to over five hundred persons, who were exposed to the disease in waiting on the sick, and not one of them took it. The writer further said that he considered this sufficient evidence that the administration of dilute vaccine virus was a perfect prophylactic and that this is the proper way to use it.

I have in my possession a letter from Dr. Fincke of Brooklyn, N. Y., in which he refers Dr. C. A. Beldin, who experienced the prophylactic action of Variolinum$^{m}$ on himself, having on hand a confluent case of small-pox, and many other cases besides, in 1892. He got similar symptoms to small-pox for one day, but next day he was free from it and able to attend to his practice without being infected. Dr. Fincke says: "In 1893 he asked for another vial of Variolin.$^{m}$ (which he preferred), with the following words: 'I am using it altogether instead of vaccinating. I refuse to vaccinate. More are killed and maimed in some way for life by vaccination than die of small-pox. I have had cases that had been exposed to small-pox by being with small-pox cases for nine days. I gave them Variolin.$^{m}$, and it got ahead of the small-pox and aborted it.'"

Dr. Fincke also says, "I do not vaccinate any more for the last twenty years, and give Variolin.$^{900}$ as a prophylactic. I never heard that any one so protected had been affected by small-pox. This spring I gave to a patient of mine Variolin.$^{900}$, whose sister got small-pox through having been vaccinated two weeks before without effect, and his little brother, an infant, had varioloid at the same time. But though in contact with them he was not infected."

Dr. Fincke relies on the single dose, but I have given one dose per day for three successive days. During the past winter all my patients who applied for vaccination have accepted Variolin. in preference, and others who were not my regular patients applied for it.

Aside from the children of the small-pox patients who received Vac.$^{30}$, I do not know whether any of the persons who received Variolin. have been exposed to small-pox.

I believe that in case of an epidemic of small-pox, the remedy that corresponds to the epidemic would be the best prophylatic, be that remedy what it may.

